# Vitamin A and Bone Health: A Review on Current Evidence

**DOI:** 10.3390/molecules26061757

**Published:** 2021-03-21

**Authors:** Michelle Min Fang Yee, Kok-Yong Chin, Soelaiman Ima-Nirwana, Sok Kuan Wong

**Affiliations:** Department of Pharmacology, Faculty of Medicine, University Kebangsaan Malaysia, Jalan Yaacob Latif, Bandar Tun Razak, Cheras 56000, Kuala Lumpur, Malaysia; michelleyeeminfang@gmail.com (M.M.F.Y.); chinkokyong@ppukm.ukm.edu.my (K.-Y.C.); imasoel@gmail.com (S.I.-N.)

**Keywords:** carotene, cryptoxanthin, fracture, osteoporosis, retinol

## Abstract

Vitamin A is a fat-soluble micronutrient essential for growth, immunity, and good vision. The preformed retinol is commonly found in food of animal origin whereas provitamin A is derived from food of plant origin. This review summarises the current evidence from animal, human and cell-culture studies on the effects of vitamin A towards bone health. Animal studies showed that the negative effects of retinol on the skeleton were observed at higher concentrations, especially on the cortical bone. In humans, the direct relationship between vitamin A and poor bone health was more pronounced in individuals with obesity or vitamin D deficiency. Mechanistically, vitamin A differentially influenced the stages of osteogenesis by enhancing early osteoblastic differentiation and inhibiting bone mineralisation via retinoic acid receptor (RAR) signalling and modulation of osteocyte/osteoblast-related bone peptides. However, adequate vitamin A intake through food or supplements was shown to maintain healthy bones. Meanwhile, provitamin A (carotene and β-cryptoxanthin) may also protect bone. In vitro evidence showed that carotene and β-cryptoxanthin may serve as precursors for retinoids, specifically all-trans-retinoic acid, which serve as ligand for RARs to promote osteogenesis and suppressed nuclear factor-kappa B activation to inhibit the differentiation and maturation of osteoclasts. In conclusion, we suggest that both vitamin A and provitamin A may be potential bone-protecting agents, and more studies are warranted to support this hypothesis.

## 1. Introduction

Bone health is maintained through normal bone remodelling, which is an active and dynamic process whereby the activities of bone resorption and formation occur in balance to maintain bone microarchitecture, strength, and mineral homeostasis. Osteoblasts are mainly involved in bone deposition while osteoclasts are responsible for bone resorption [1]. Various endogenous (hormones, growth factors, cytokines) and exogenous (nutrients, drugs, pollutants) factors can influence this delicately orchestrated physiological process. Imbalance of any of these factors could result in dysregulated bone remodelling, which favours bone loss [2,3,4].

Vitamin A has been extensively studied for its role in bone health. Vitamin A can be consumed in two forms, i.e., preformed retinol and provitamin A. Preformed retinol is often found in food originated from animals, such as dairy, liver and eggs. Provitamin A, such as alpha (α)-carotene, beta (β)-carotene or β-cryptoxanthin, are commonly found in plant-based food, such as fruits and vegetables [5]. Amongst these, the most common provitamin A is β-carotene. The consumed provitamin A can be absorbed as intact carotenoid or oxidised to retinal and subsequently reduced to retinol in the enterocyte. On the other hand, preformed retinol is directly absorbed from the intestine. Retinol is esterified to retinyl ester, packaged into chylomicrons along with the intact provitamin A, and stored in the liver. Retinyl ester is then converted to retinol and bound to retinol-binding protein (RBP) to be released into the circulation. Both retinol and provitamin A reach the peripheral cells (including bone) via signalling receptor and transporter of retinol (STRA6) and delivery by chylomicrons. Previous study indicated that STRA6 is highly expressed in human mesenchymal stem cells obtained from osteoporotic subjects [6]. In addition, bone is an important organ responsible for the clearance of chylomicron remnants. Thus, fat-soluble vitamins can be delivered to osteoblasts in vivo via chylomicrons [7]. Upon reaching the targeted cells, retinol and provitamin A undergo the conversion to all-trans-retinoic acid (the biologically active metabolite of vitamin A), followed by binding to the RAR and retinoid X receptor (RXR) heterodimers to exert their effects [8]. 

The current evidence yielded inconsistent outcomes showing positive, negative, and negligible effects of vitamin A on bone health. Carotene and β-cryptoxanthin potentially influence bone homeostasis by exhibiting stimulatory effects on osteoblastic bone formation and inhibitory effects on osteoclastic bone resorption [9,10]. Higher bone mineral density (BMD) and lower fracture risk have been reported in individuals with higher vitamin A intake [11,12]. In contrast, some studies reported that dietary vitamin A intake in the form of multivitamin supplementation or food fortification was associated with increased risk of fracture and accelerated age-related bone loss [13,14]. Others reported a lack of association between vitamin A intake and fragility fracture [15]. Short-term hypervitaminosis A also caused an acceleration of cortical bone loss in laboratory animals [16]. Besides, vitamin A has been suggested to antagonise the role of vitamin D in increasing calcium absorption and maintaining homeostatic serum calcium concentration. Both retinoic acid and 1,25-hydroxyvitamin D share a common nuclear receptor (RXR) following their interaction with RAR and vitamin D receptor (VDR), respectively. Hence, a high vitamin A concentration could reduce vitamin D function [17]. Therefore, vitamin A could exert both positive and negative impacts on bone. Thus, understanding the mechanism of action of vitamin A is important to determine its net skeletal effect. 

In this review, evidence on the effects of vitamin A and provitamin A on bone in animals were collated. This is followed by discourse on the relationship between intake/serum concentration of vitamin A/provitamin A and BMD, osteoporosis or fracture risk in humans. The mechanism of action underlying the effects of vitamin A or provitamin A was also discussed. The review will provide the readers with an overview of the relationship between vitamin A and bone health.

## 2. Effects of Vitamin A on Bone Health in Animals

Using rodent animal models, Lionikaite et al. conducted two experiments to evaluate the effects of synthetic lipid-soluble retinyl acetate on bone phenotype. In the earlier study, various doses of retinyl acetate were given to female C57BL6/J mice. The doses of retinyl acetate (20 µg/g diet) did not affect bone length, trabecular bone microstructure at vertebra, BMD and cortical bone at the tibia. A higher dose (60 µg/g diet) of retinyl acetate caused deterioration of cortical bone quality, whereby cortical bone mineral content (BMC), BMD, cortical thickness (Ct.Th), periosteal perimeter (Ps.Pm), endocortical perimeter (Ec.Pm), polar moment of inertia, marrow area (Ma.Ar), and total area (Tt.Ar) were reduced. The expressions of osteocalcin (OCN) and alkaline phosphatase (ALP) were also decreased following supplementation of retinyl acetate at 60 µg/g diet. For animal model with hypervitaminosis A, retinyl acetate at the dose of 450 µg/g diet was provided to animals for eight days. Significant increases in osteoclast number (Oc.N) and expression of osteoclastogenic genes, such as acid phosphatase 5 (ACP5), cathepsin K (CTSK), receptor activator of nuclear factor kappa-B ligand (RANKL) and tartrate-resistant acid phosphatase (TRAP) were observed, along with reductions in cortical bone architecture [16]. In the subsequent study, they tested the effects of retinyl acetate on bone using the experimental mouse model with and without tibial loading. The animals were subjected to axial loading at tibia on alternate days for two weeks to induce bone formation after feeding the animals with a diet containing retinyl acetate (60 µg/g diet) for four weeks. Retinyl acetate supplementation suppressed the loading-induced increases in bone volume/total volume (BV/TV), trabecular number (Tb.N), cortical area (Ct.Ar), Ma.Ar, Ct.Th, Ec.Pm, Ps.Pm, bone formation rate (BFR), mineral apposition rate (MAR) and loading-induced decrease in trabecular separation (Tb.Sp). Intake of retinyl acetate through diet also reduced the expression of osteoblastic genes, including osterix (OSX), ALP, and type 1 collagen (COL1). For control animals not subjected to tibial loading, retinyl acetate supplementation induced no changes in trabecular bone microstructure and osteogenic gene expression. However, retinyl acetate supplementation promoted BFR, mineralising surface (MS) and MAR at endocortical region but decreased Ct.Ar, Ma.Ar, Ec.Pm, and Ps.Pm [18]. In another study involving mature female Sprague Dawley rats, retinyl palmitate (natural alternative of retinyl acetate) and retinyl acetate were enriched in the diet (120 or 600 IU/g pellet) and given to the animals for 12 weeks. Findings showed that serum retinyl esters, indicators of vitamin A intoxication, were elevated after supplementation. The animals fed on the highest dose of retinyl palmitate and retinyl acetate had reduced humerus diameter, Ps.Pm, and total cross-sectional area. No alteration was detected in humerus length, Ec.Pm, and BMD. Together with the bone phenotype changes, the serum levels of other fat-soluble vitamins (vitamin D and E) were reduced [19]. 

Several important points can be concluded from all the studies that use preformed retinol. First, preformed vitamin A at a lower dose did not confer any side effects on bone. Second, the supplementation of vitamin A might exert negligible or fewer negative effects on normal animals. Third, the clinically relevant dosage of vitamin A might also suppress bone formation stimulated by external factors (such as mechanical loading). Fourth, the adverse event of vitamin A at high dose might be more pronounced at cortical bone rather than trabecular bone, mainly through its action on osteoclasts. The effects of vitamin A on bone in vivo are summarized in Table 1. 

## 3. Effects of Provitamin A on Bone Health in Animals

Limited animal studies have been conducted to identify the bone-protecting effects of provitamin A (Table 1). A recent study by Matsumoto et al., demonstrated that β-carotene prevented bone loss in a mouse model of hindlimb unloading. In this study, female ddY mice were subjected to unloading using tail suspension method whereby the hindlimbs of mice were elevated to produce 30° head-down tilt which results in cephalad fluid shift and avoids weight-bearing by hindquarters [21]. The unloaded animals were fed with diet containing β-carotene (0.025–0.25%) for three weeks. The unloaded animals treated with 0.025% β-carotene had higher BMD at whole and proximal tibia than the negative controls. No changes were observed in bone strength as well as expression of osteogenic and osteoclastogenic genes [20]. Based on the findings obtained from this study, β-carotene potentially prevents bone loss in a dose-independent manner. In addition, better outcomes may be observed with longer treatment duration. Further investigations using various animal models of bone loss are recommended to validate the bone-sparing action of β-carotene.

## 4. Effects of vitamin A on Bone Health in Humans 

The relationship between vitamin A intake, serum vitamin A concentration, and bone health in humans has been extensively reported with heterogeneous findings. Literature indicating positive, negative, or negligible effects of vitamin A on bone are available (Table 2). 

### 4.1. Positive Effect

The protective effects of vitamin A on bone were reported in several cohorts, case–control, and cross-sectional studies. An earlier cross-sectional study (in postmenopausal Korean women population) by Choi et al., showed that vitamin A intake was lower in subjects with osteopenia and osteoporosis compared to the normal subject, suggesting a positive association between vitamin A intake and BMD in elderly women aged 65 to 80 years old [22]. In the Korea National Health and Nutrition Examination Survey (KNHANES) consisting of 2907 men and 3574 women (aged ≥ 50 years), the researchers found that dietary intake of vitamin A was positively correlated with BMD at the total hip and femoral neck in men as well as at lumbar spine in women. Interestingly, this positive relationship was only found in individuals with moderate and high levels of vitamin D, but not in subjects with vitamin D deficiency [12]. In the subsequent year, Kim and colleagues reported a positive correlation between T-score of the lumbar spine, femoral neck, and total hip with vitamin A intake among Korean postmenopausal women [23]. A case–control study by Sun et al., included participants who were newly diagnosed with hip fractures. The findings showed that a moderate to high dietary intake of animal-derived vitamin A was associated with reduced hip fracture risk [24]. In addition, Karamati et al., studied the effect of different nutrient patterns on BMD among postmenopausal Iranian women. They found that intake of food abundant in vitamin A, β-carotene, folate, fibre, vitamin B6, potassium, vitamin C, vitamin K, magnesium, copper and manganese had significant positive association with lumbar BMD [25]. The Rancho Bernardo Heart and Chronic Disease Study comprising elderly men and postmenopausal women aged 55 years old and above demonstrated an inverse U-shaped relationship between retinol intake and BMD. Initially, increasing retinol intake (in the range of 0–2000 IU) was associated with an increase in BMD. After BMD reached the peak at retinol intake 2000–2800 IU, BMD decreased with further increases in retinol intake [26]. In the prospective population-based Rotterdam Study on Dutch subjects aged 55 years and above, higher dietary intake of total vitamin A increased BMD and lowered fracture risk. They also found a favourable relationship between high vitamin A intake and fracture risk in overweight subjects [11]. In the prospective cohort by Chen and co-researchers, a positive association between dietary consumption or serum level of retinol and BMD at various bone sites was observed in Chinese men and women [27].

### 4.2. Negative Effect 

Other studies revealed the negative effects of vitamin A intake on skeletal system. Melhus et al., observed a negative association between retinol intake and BMD (in a randomly selected female population). Increased dietary retinol intake was associated with reductions in BMD at femoral neck, Ward’s triangle, trochanter region of proximal femur, lumbar spine and total body. Similarly, hip fracture risk was doubled with every unit increase of retinol intake [28]. A cross-sectional study among Brazilian postmenopausal women with osteoporosis demonstrated that subjects with lower vitamin A intake had higher lumbar spine BMD. However, this inverse association between vitamin A and BMD could be attenuated by concurrent intake of other antioxidants [29]. Based on data from the Spanish postmenopausal women, approximately 60% of women with high serum retinol level was vitamin D deficient. The risk of osteoporosis was higher in postmenopausal Spanish women with the highest retinol quintile, reiterating that both high retinol levels and vitamin D deficiency are important risk factors for osteoporosis [30]. In a cross-sectional study involving untreated osteoporotic postmenopausal women (aged <65 years), a higher serum retinol level was associated with lower BMD at the spine and hip [14]. In a prospective cohort study among postmenopausal women between 55 to 69 years old, a negative effect of vitamin A supplementation on bone was detected. Vitamin A supplement users had a higher risk of hip fracture than non-users [31]. A study which enrolled postmenopausal women (aged 34 to 77 years old) in the Nurses’ Health Study pointed out that the intake of vitamin A or retinol from food source only or from food plus supplement was positively associated with increased risk of hip fracture, in which this correlation was attenuated in women prescribed with oestrogen. In this study, consumption of vitamin A or retinol via supplements exerted more harmful effects to the bone rather than dietary intake [32]. Several years later, the same group of researchers observed increased osteoporosis risk in postmenopausal women with higher retinol levels. In this study, mean serum retinol level in osteoporotic postmenopausal women was higher than postmenopausal women with osteopenia and normal BMD [33]. In the Southampton Women’s Study cohort, maternal serum retinol in late pregnancy was negatively associated with offspring’s total body BMC and bone area [34]. In a longitudinal study by Caire-Juvera et al., involving postmenopausal women aged 50 to 79 years, the fracture risk was greater in the highest quintile of total vitamin A intake compared to the lowest quintile. However, such a relationship was only apparent in women with low vitamin D [13]. Additionally, in a study by Michaelsson et al., involving 2322 men (in Uppsala, Sweden), the highest fracture risk was observed in men with the highest serum retinol [35].

### 4.3. No Association 

A cross-sectional study reported the absence of a significant association between vitamin A intake and fracture due to bone frailty among Brazilian adults [15]. Vitamin A intake also showed no significant association with BMD at lumbar spine and femoral neck among perimenopausal women in a Danish population [36]. A study by Johansson and Melhus found that retinyl palmitate intake did not alter C-telopeptide of type 1 collagen (CTX), a marker of bone resorption, in healthy adults. However, intake of retinyl palmitate alone decreased serum calcium. This response was diminished with combined intake of retinyl palmitate and vitamin D [17]. Among men and women enrolling in a cancer prevention program, there was no association between plasma retinol level and risk of any fracture [37]. The third National Health and Nutrition Examination Survey (NHANES III) also showed that fasting serum retinyl ester concentration was not correlated with BMD at any site, increased risk or presence of osteopenia or osteoporosis [38]. Serum retinol concentration and retinol-binding protein 4 (RBP4) were also not associated with osteoporosis risk among Thai postmenopausal women. Although significant association was not found in this study, the authors pointed out the positive relationship between transthyretin (TTR, a prealbumin that indirectly transports retinol) and BMD. The level of TTR in serum was lower, indicating mild malnutrition in osteoporotic subjects [39]. Results obtained from a prospective cohort indicated no association between vitamin A intake from food and the risk of hip and all fractures [31]. There was no significant interaction between serum retinol concentration and hip fracture risk in community-dwelling older Norwegians [40]. In the subsequent year, Kawahara and colleagues concluded that consumption of tablet containing 7576 µg of retinol palmitate for six weeks caused no changes in bone-specific ALP, N-telopeptide of type 1 collagen (NTX), and OCN in healthy men and placebo-treated group. [41].

Taken together, the current state of evidence describes the inconsistent findings on the relationship between the intake or circulating level of vitamin A on bone. The discrepancy obtained from these studies may be attributed to the variation in study design, population, duration, subject’s age, gender, and health condition. However, there are several considerations to be acknowledged. Firstly, vitamin A intake may not adversely affect bones if adequate serum vitamin D concentration is maintained. Vitamin D deficiency in subjects with high vitamin A level may be an overlooked contributing factor for bone loss, emphasising the need for evaluation of vitamin A and D status in future studies for better understanding of the relationship between vitamin A and bone health. Secondly, vitamin A may be prone to cause deterioration of bone mass in overweight or obese individuals. Thirdly, the potential negative effect of vitamin A on bone may be antagonised by intake or supplementation of other antioxidants or bone-sparing agents (such as oestrogen). High levels of antioxidant in the body inhibit the overproduction of reactive oxygen species (ROS), favouring osteoblast differentiation, mineralisation, and inhibition of osteoclastogenic activity. Oestrogen also stimulates osteoblast differentiation and osteoclast apoptosis as well as inhibiting osteoblast apoptosis and osteoclast differentiation. Thus, these factors should not be neglected in elucidating the association between vitamin A and bone health.

## 5. Effects of Provitamin A on Bone Health in Humans

A positive relationship was detected between BMD at various sites (including lumbar spine and femoral neck) and dietary β-carotene intake among postmenopausal women in Korea [23] and Iranian populations [25]. Vegetable-derived β-carotene intake was also related to reduced hip fracture in the Chinese elderly [24]. Two research groups recently explored the relationship between dietary provitamin A (carotene and β-cryptoxanthin) intake and bone health. Postmenopausal women with daily β-carotene and β-cryptoxanthin in the highest quintile had a lower risk of osteopenia at the lumbar spine and total hip. Higher β-carotene and β-cryptoxanthin intakes were also associated with higher femoral neck, total hip, and whole-body BMD in men and postmenopausal women [42]. A study by Cao et al., showed that higher α-carotene, β-carotene and β-cryptoxanthin intake was inversely correlated with risk of hip fracture [43]. In Chinese men and women with a greater serum β-carotene level, higher BMD values at the whole body, lumbar spine and femoral neck were detected [27]. In line with previous studies, another group of researchers found a positive association between maternal serum β-carotene level and neonatal total BMC and bone area [34]. Results obtained from men and women participating in a cancer prevention programme showed an inverse relationship between plasma total carotene concentration and fracture risk [37]. A comprehensive observational study by Maggio et al., depicted lower α- and β-carotene levels in plasma in osteoporotic subjects relative to control subjects, suggesting a positive association between carotene level and bone health [44]. Zhang et al., reported similar findings in their cross-sectional study, whereby a higher circulating level of α-carotene was associated with a higher BMD value for the whole body and hip regions [45]. 

On the contrary, some studies displayed no association between provitamin A intake or serum concentration with bone health. Intake of β-carotene did not contribute significantly to the fracture risk in women aged 34 to 77 years old [32]. In the following year, a population-based longitudinal study did not find a relationship between serum β-carotene level and fracture risk in healthy men [35]. Recent research conducted in Dutch subjects aged 55 years old and above also found no interaction between dietary β-carotene intake and BMD [11]. 

In summary, most studies demonstrated the protective effects of provitamin A on bone, except three studies showed no association. Likewise, the inconclusive findings may be due to the distinct study design and heterogeneity of subjects recruited in the studies. Other confounding factors should be considered, such as vitamin D status, lifestyle, antioxidant intake and medications, that could potentially affect bone metabolism. Moreover, carotene may exert a negligible effect on bone in healthy individuals. 

## 6. The Potential Underlying Mechanisms of Vitamin A and Provitamin A 

Mesenchymal stem cells (MSCs) are a population of stromal cells present in the bone marrow and most connective tissues, capable of differentiation into mesenchymal tissues such as bone and cartilage. During osteogenic differentiation, the expression of early markers (ALP and COL1) is initiated, followed by the production of late markers [OCN and osteopontin (OPN)]. The osteogenetic process is controlled by Runt-related transcription factor-2 (Runx-2), a key signalling molecule orchestrating multiple osteoblastogenic signals. The interaction of bone morphogenetic protein-2 (BMP-2) and various growth factors with their respective receptors activate Runx-2, which in turn amplifies the transcriptional activity of its own and other osteogenic markers [46]. 

The regulation of bone peptides produced by osteocyte and/or osteoblast plays an important role in bone metabolism. Sclerostin (SOST) and Dickkopf-related protein 1 (DKK1) negatively regulates the canonical Wingless (Wnt)/beta (β)-catenin pathway, a crucial pathway directing the commitment of mesenchymal stem cells into the osteoblastic lineage and its subsequent differentiation [47]. Phosphate regulating endopeptidase homolog X-linked (Phex) and dentin matrix protein 1 (DMP1) negatively regulate the expression of fibroblast growth factor-23 (FGF-23), a potent regulator for phosphate and vitamin D metabolism, thus suggesting their role in bone mineralisation [48,49]. Specifically, a higher level of FGF-23 suppresses reabsorption of phosphate in the kidney and increases excretion of phosphate in the urine. FGF-23 also inhibits 1α-hydroxylase, diminishing the formation of calcitriol [1,25(OH)2D3, the active form of vitamin D [50]. In osteoclastogenesis, osteoprotegerin (OPG) and RANKL are the main components regulating the differentiation of osteoclast precursors into mature osteoclasts [51]. The interaction of RANKL with its receptor, receptor activator of nuclear factor kappa-B (RANK), recruits tumour necrosis factor receptor-associated factor 6 (TRAF6) and activates a series of downstream events [such as nuclear factor-kappa B (NF-κB), phosphatidylinositol 3-kinase (PI3K)/protein kinase B (Akt), mitogen-activated protein kinase (MAPK), and nuclear factor of activated T-cells cytoplasmic 1 (NFATc1)] essential for expression of osteoclastogenic genes [52]. Meanwhile, OPG acts as a decoy receptor for RANKL preventing the RANK-RANKL interaction. 

Treatment with retinol or retinoic acid at the concentration range from 1 to 100 nM stimulated the differentiation of murine pre-osteoblastic (MC3T3-E1) cells by raising ALP activity and OPN expression [53]. Nonetheless, Lind et al., found that retinoic acid was a negative regulator for mineralisation. Retinoic acid at 4 or 400 nM reduced calcium deposition in primary human osteoblasts. In MC3T3-E1 cells, cell proliferation and osteogenic gene expression (including ALP, OCN, Runx-2, and OSX) were inhibited after incubation with retinoic acid at higher concentrations. Treatment with high-concentration retinoic acid also caused the downregulation of Phex, SOST, and FGF-23 but upregulation of RANKL and DMP1 at osteoblast level. Administration of CYP26B1 (a major enzyme for retinoic acid degradation) inhibitor increased endogenous retinoic acid and caused bone demineralisation. The inhibitory action of retinoic acid at high concentrations on bone homeostasis is exerted through RAR signalling activation. As evidence, the adverse effects of retinoic acid on osteoblast mineralisation were prevented by administering a pan-RAR antagonist [54]. Theoretically, vitamin A promotes osteoblast differentiation but inhibits bone mineralization at low concentration. High concentration of preformed vitamin A exerts net detrimental effects on both osteoblast differentiation and mineralization via its concerted effects of osteogenic gene inhibition, osteoclastogenic gene activation, and modulation of osteocyte/osteoblast-related bone peptides. 

**Figure 1:**
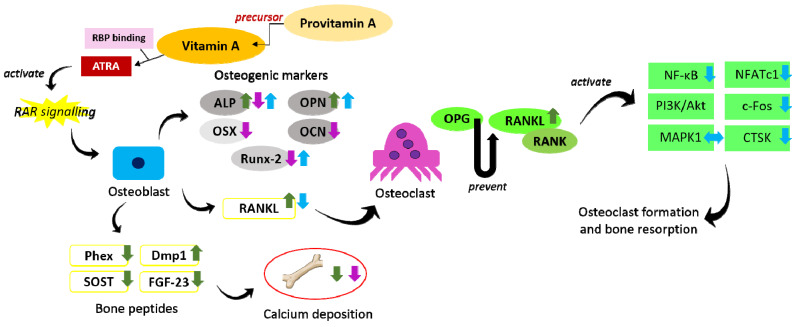
The effects of vitamin A and provitamin A on the regulation of osteogenesis and osteoclastogenesis. Provitamin A serves as the precursor for vitamin A, which is then converted to ATRA in target cells to act as ligand for RAR and perform their functions. Vitamin A at low concentration promotes osteoblastic activity but inhibits bone mineralisation (indicated by green arrows). Vitamin A at high concentration inhibits both bone differentiation and bone mineralisation (indicated by purple arrows). Provitamin A promotes osteoblast differentiation and inhibits osteoclastic activity (indicated by blue arrows). The arrow pointing upward indicates an increase, the arrow pointing downward indicates a decrease, and the two-headed arrow indicates no change. Abbreviations: Akt = protein kinase B; ALP = alkaline phosphatase; ATRA = all-trans-retinoic acid; c-Fos = Fos proto oncogene; CTSK = cathepsin K; Dmp1 = dentin matrix protein 1; FGF-23 = fibroblast growth factor-23; MAPK = mitogen-activated protein kinase; NFATc1 = nuclear factor of activated T-cells cytoplasmic 1; NF-κB = nuclear factor-kappa B; OCN = osteocalcin; OPG = osteoprotegerin; OPN = osteopontin; OSX = osterix; PI3K = phosphatidylinositol 3-kinase; Phex = phosphate regulating endopeptidase homolog X-linked; RANK = receptor activator of nuclear factor kappa-B; RANKL = receptor activator of nuclear factor-kappa B ligand; RAR = retinoic acid receptor; RBP = retinol-binding protein; Runx-2 = runt-related transcription factor 2; SOST = sclerostin.

For provitamin A, the combination of β-carotene with isoflavones increased cell growth, ALP, Runx-2, and OPN expression on MC3T3-E1 cells. The induction of early osteoblastic differentiation by β-carotene was mediated through RAR signalling [55]. Using primary osteoblastic cells derived from mouse calvariae and stimulated with lipopolysaccharides, treatment with β-cryptoxanthin inhibited the gene expression of cyclooxygenase-2 and membrane-bound prostaglandin E synthase, leading to reduced synthesis of prostaglandin E2 and subsequent suppression of RANKL expression [9]. To investigate the effects of provitamin A on osteoclastogenesis, bone marrow-derived monocytes/macrophages were stimulated by RANKL and treated with β-carotene (0.4–0.6 µM). β-carotene decreased the viability of these cells, reduced density of TRAP-positive areas, osteoblast numbers and resorption pit formation, as well as increasing lactate dehydrogenase (LDH) release (an indicator of cell apoptosis). Attenuation of NF-κB activation was observed, but no effect on MAPK pathway was observed after β-carotene administration. In addition, downregulation of NFATc1, Fos proto-oncogene (c-Fos), and CTSK were detected [10]. In RAW264.7 cells stimulated by RANKL, β-cryptoxanthin suppressed the formation of osteoclast-like cells, evidenced by reductions in TRAP-positive multinucleated cells, CTSK expression and NF-κB activation [9]. In short, provitamin A potentially protects the bone by enhancing osteoblast differentiation and inhibiting osteoclastic activity. However, several research gaps remain to be filled. The individual effect of β-carotene on osteoblast differentiation, the action of β-carotene and β-cryptoxanthin on bone mineralisation remain to be validated. The effects of vitamin A and provitamin A on bone cells have been summarised in Table 3. The postulated skeletal action of vitamin A and provitamin A is depicted in Figure 1. 

## 7. Conclusions

Adequate vitamin A intake either from diet or supplementation is necessary to maintain bone health. Based on the in vitro evidence, the skeletally active concentration of vitamin A and provitamin A ranges from 1 to 100 nM and 0.1 to 10 μM, respectively. In animals, the recommended dosage for vitamin A is less than 60 μg/g. Limited study has been conducted on provitamin A on bone health in vivo, thus its optimum dose remains to be elucidated. In humans, the recommended daily allowance (RDA) of vitamin A is 900 µg (3000 IU) and 700 µg (2330 IU) for men and non-pregnant, non-lactating women, respectively [56]. At these doses, vitamin A may be protective to the skeleton. Hypervitaminosis A causes bone problems, especially in individuals with insufficient vitamin D. As such, hypervitaminosis A causes low BMD and increases fracture risk because retinoic acid inhibits osteoblast differentiation and mineralisation at high doses. Provitamin A seems to be beneficial to the bone by stimulating osteoblastic activity and bone formation as well as inhibiting osteoclastic activity and bone resorption. These findings await further validation using healthy animals and various established in vivo osteoporotic and fracture models. However, the possible harmful effects of high dose provitamin A on bone should not be neglected. 

## Figures and Tables

**Table 1:** Summary on the effects of vitamin A and provitamin A on bone health in animals.

Type of Model	Treatment, Dose, Duration	Findings	Reference
Female C57BL6/J mice (aged 9–19 weeks)	Retinyl acetate (20 µg/g diet; 4 or 10 weeks)	No changes in femur length, tibia length, and BMD at tibia No changes in BV/TV, Tb.N, Tb.Th and Tb.Sp at vertebra No changes in cortical BMC, BMD, Ct.Th, Ec.Pm, Ps.Pm, polar moment of inertia	[16]
Female C57BL6/J mice (aged 9–19 weeks)	Retinyl acetate (60 µg/g diet; 4 or 10 weeks)	No changes in femur length, tibia length, and BMD at tibia No changes in, BV/TV, Tb.N, Tb.Th and Tb.Sp at vertebra ↓ cortical BMC, BMD, Ct.Th, Ec.Pm, Ps.Pm, polar moment of inertia ↓ OCN, ALP at tibial cortical bone ↑ endocortical MAR and BFR ↓ Ma.Ar and Tt.Ar	
Female C57BL6/J mice (n = 10/group; aged 8–9 weeks)	Retinyl acetate (450 µg/g diet; 8 days)	↓ endocortical circumference, periosteal circumference, cortical BMC, and Ct.Th ↑ Oc.N at periosteum and ↓ Oc.N at endosteum ↑ Acp5, CTSK, RANKL and TRAP in the cortical bone of tibia	
Female C57BL/6N mice subjected to tibia loading (n = 8/group; aged 12–13 weeks)	Retinyl acetate (60 µg/g diet; 4 weeks)	↓ BV/TV, Tb.N, Ct.Ar, Ma.Ar, Ct.Th, Ec.Pm, Ps.Pm, BFR, MS, and MAR; ↑ Tb.Sp ↓ OSX, ALP, COL1 and no change in sclerostin	[18]
Female C57BL/6N mice subjected to tibia unloading (n = 8/group; aged 12–13 weeks)	Retinyl acetate (60 µg/g diet; 4 weeks)	No changes in BV/TV, Tb.Th, Tb.N, Tb.Sp ↓ Ct.Ar, Ma.Ar, Ec.Pm and Ps.Pm; no change in Ct.Th ↑ BFR, MS and MAR at endocortical but no changes at periosteal No change in OSX, ALP, COL1, and sclerostin	
Mature female Sprague- Dawley rats (n = 45, 15/group, aged 3 months)	Retinyl palmitate and retinyl acetate (600 IU/g diet; 12 weeks)	No change in length of humerus, endocortical circumference and BMD. ↓ humerus diameter, Ps.Pm, total cross-sectional area ↑ serum retinyl esters and total amount of liver retinoid ↓ serum vitamin D and E concentrations	[19]
Female ddY mice subjected to hindlimb unloading (n = 6-8)	β-carotene (0.025%, 3 weeks)	↑ whole and proximal tibia BMD No changes in minimum and polar moment of inertia of cross-sectional areas. No changes in ALP, OSX, RANKL, OPG, and RANKL/OPG ratio.	[20]

List of abbreviations: Acp5: acid phosphatase 5; ALP: alkaline phosphatase; BFR: bone formation rate; BMC: bone mineral content; BMD: bone mineral density; BV/TV: bone volume per tissue volume; COL1: type 1 collagen; Ct.Ar: cortical bone area; CTSK: Cathepsin K; Ct.Th: cortical thickness; Ec.Pm: endocortical perimeters; Ma.Ar: marrow area; MAR: mineral apposition rate; MS: mineralising surface; OCN: osteocalcin; Oc.N: osteoclast number; OPG: osteoprotegerin; OSX: osterix; Ps.Pm: periosteal perimeter; RANKL: receptor activator of nuclear factor-kappa B ligand; Tb.N: trabecular number; Tb.Sp: trabecular separation; Tb.Th: trabecular thickness; TRAP: tatrate-resistant acid phosphatase; Tt.Ar: total area.

**Table 2:** Summary on the effects of vitamin A and provitamin A on bone health in humans.

Study Design	Study Population	Vitamin A Intake/Concentration	Findings	Reference
Cross-sectional study	Elderly women (n = 101, aged 65–80 years)	Vitamin A intake	Vitamin A intake was lower in the osteopenia and osteoporosis group than control.	[22]
Cross-sectional study	Subjects participating in KNHANES between 2008–2011 (n = 6481; aged ≥ 50 years)	Vitamin A intake	Dietary vitamin A was positively associated with total hip and femoral neck BMD in men and lumbar spine BMD in women with high serum vitamin D.	[12]
Cross-sectional study	Postmenopausal women (n = 189, aged 50-75 years)	Vitamin A intake	Vitamin A intake was positively associated with T-score of lumbar spine, femoral neck, and total hip.	[23]
Case–control study	Elderly Chinese newly diagnosed with hip fractures and control participants in 2009–2013 (n = 1452)	Vitamin A intake	Dietary intake of vitamin A [OR = 0.37 (95% CI 0.28–0.50)] was negatively associated with hip fracture risk.	[24]
Cross-sectional study	Postmenopausal women (n = 160, aged 50–85 years)	Dietary pattern high in vitamin A and other nutrients	Dietary pattern high in vitamin A [OR = 0.08 (95% CI 0.02–0.15)] was positively associated with BMD at lumbar spine.	[25]
Population-based cohort study	Men and postmenopausal women participating in Rancho Bernardo Heart and Chronic Disease Study between 1988–1992 (n = 1526; aged ≥55 years)	Retinol intake	Analogous inverse U-shaped association was observed between retinol intake and BMD. Retinol intake at 0–2000 IU was positively associated with BMD but negatively associated with BMD after 2800 IU	[26]
Prospective, population-based cohort study	Dutch subjects participating in Rotterdam Study between 1990–1993 (n = 5288; aged ≥55 years)	Total vitamin A and retinol intake	Dietary total vitamin A and retinol intake was positively associated with BMD. Dietary total vitamin A [HR = 0.82 (95% CI 0.69–0.97)] and retinol [HR retinol = 0.81 (95% CI 0.68–0.96)] intake was negatively associated with fracture risk. High vitamin A intake and fracture risk only in overweight subjects.	[11]
Prospective cohort study	Chinese men and women recruited between 2008–2010 (n = 3169; aged 40–75 years)	Dietary consumption and serum level of retinol	Dietary intake of retinol was positively associated with BMD at total hip and femoral neck Serum levels of retinol and β-carotene-to-retinol ratio were positively associated with BMD at various sites.	[27]
Cross-sectional study and nested case–control study	Cross-sectional study: women (n = 175; aged 28–74 years) Nested case–control study: women who had first hip fracture within 2–64 months after enrolment (n = 247; aged 40–76 years) and age-matched control (n = 873; aged 40–76 years)	Retinol intake	Retinol intake was negatively associated with BMD (at femoral neck, ward triangle, trochanter region of the proximal femur, lumbar spine, total body) and positively associated with hip fracture risk [OR = 1.54 (95% CI 1.06–2.24)].	[28]
Cross-sectional study	Postmenopausal women with osteoporosis attending specialised outpatient clinic of UNIFESP between 2009–2012 (n = 150, aged ≥ 45 years)	Vitamin A intake from food	Vitamin A intake was negatively associated with lumbar spine BMD.	[29]
Cross-sectional study	Healthy postmenopausal Spanish women from breast cancer screening program (n = 229; aged 57.4 ± 6.4 years)	Serum retinol concentration	Serum retinol level was positively associated with risk of osteoporosis.	[30]
Cross-sectional study	Non-treated osteoporotic postmenopausal women (n = 154; aged ˃65 years)	Serum retinol concentration	Higher retinol was associated with lower BMD at lumbar spine and femoral neck.	[14]
Prospective cohort study	Postmenopausal women participating in the Iowa Women Health Study in 1986 (n = 41,836; aged 55–69 years)	Vitamin A and retinol intake from supplement	Vitamin A [RR = 1.22 (95% CI 0.98–1.52)] and retinol [RR = 1.24 (95% CI 0.96–1.59)] supplement users had higher hip fracture risk compared to non-users.	[31]
Prospective cohort study	Postmenopausal women in the Nurses’ Health Study (n = 72,337; aged 34–77 years)	Vitamin A intake from food and supplement	Vitamin A [RR = 1.82 (95% CI 0.97–3.40)] and retinol [RR = 1.69 (95% CI 1.05–2.74)] intake from food source only were positively associated with risk of hip fracture. Vitamin A [RR = 1.48 (95% CI 1.05–2.07)] and retinol [HR = 1.89 (95% CI 1.33–2.68)] intake from food plus supplement were positively associated with risk of hip fracture.	[32]
Prospective population-based cohort study	Healthy postmenopausal Spanish women from breast cancer screening program (n = 229; aged 57.4 6.4 years)	Serum retinol concentration	Serum retinol level was negatively associated with BMD at lumbar spine and total hip Serum retinol level was positively associated with risk of osteoporosis [OR = 8.37 (95% CI 2.51–27.9)].	[33]
Prospective cohort study	Non-pregnant women participating in Southampton Women’s Survey between 1998–2007 (n = 12,583; aged 20–34 years)	Maternal serum retinol concentration	Maternal serum retinol in late pregnancy was negatively associated with offspring total body BMC and bone area but not BMD or size-corrected BMC.	[34]
Longitudinal study	Postmenopausal women participating in Women’s Health Initiative Observational Study between 1993-1998 (n = 75,747; aged 50–79 years)	Total vitamin A and retinol intake from diet and supplement	Total vitamin A [HR = 1.19 (95% Cl 1.04–1.34)] and retinol intake [HR = 1.15 (95% CI 1.03–1.29)] was positively associated with fracture risk. High total vitamin A and retinol intakes were associated with fracture risk with low vitamin D intake.	[13]
Population based longitudinal study	Men (n = 2322; aged 49–51 years)	Serum retinol concentration	Serum retinol level was positively associated with the risk of fracture [rate ratio = 1.26 (95% CI 1.13–1.41)].	[35]
Cross-sectional study	Brazilian adults participating in the Brazilian Osteoporosis Study (n = 2344; aged ≥ 40 years)	Vitamin A intake	No association between vitamin A intake and presence of fractures due to bone frailty.	[15]
Cross-sectional study	Perimenopausal women (n = 1869; aged 45–58 years)	Vitamin A, retinol and β-carotene intake	No association between vitamin A, retinol, β-carotene intake and BMD at lumbar spine and femoral neck.	[36]
Cross-sectional study	Healthy subjects (n = 9; aged 24–41 years)	Retinyl palmitate, or 1,25(OH)_2_D_3_ or both combined	All treatment did not affect bone resorption. Retinyl palmitate reduced serum calcium, but the combination of retinyl palmitate and 1,25(OH)_2_D_3_ increased serum calcium.	[17]
Cross-sectional study	Men and women participating in a cancer prevention programme between 1990–1996 (n = 998)	Plasma retinol concentration	Plasma retinol concentration was not associated with risk of any fracture [HR = 0.86 (95% CI 0.65–1.14)] or osteoporotic fracture [HR = 0.97 (95% CI 0.66–1.43)].	[37]
Cross-sectional study	Male and non-pregnant female participating in the NHANES III between 1988–1994 (n = 5790; aged ≥ 20 years)	Serum retinyl esters concentration	Fasting serum retinyl ester concentration was not associated with BMD. Fasting serum retinyl ester concentration was not associated with risk and presence of osteopenia/osteoporosis in men [OR = 0.99 (99% CI 0.97–1.03)], pre-menopausal [OR = 1.01 (99% CI 0.97–1.06)], and postmenopausal women [OR = 0.99 (99% CI 0.95–1.04)].	[38]
Cross-sectional study	Thai postmenopausal women with or without osteoporosis (n = 144; aged ˃ 50 years)	Serum TTR, RBP4 and retinol levels	Serum RBP4 [OR = 0.774 (95% CI 1.80–3.32)] and retinol [OR = 0.774 (95% CI 1.80–3.32)] levels was not associated with risk of osteoporosis. Serum TTR was negatively associated with risk of osteoporosis [OR = 0.119 (95% CI 0.027–0.527)]. Serum TTR level was positively associated with total radius BMD.	[39]
Prospective cohort study	Postmenopausal women participating in the Iowa Women Health Study in 1986 (n = 41,836; aged 55–69 years)	Vitamin A and retinol intake from food	Hip fracture risk was not associated with intake of vitamin A [RR = 1.08 (95% CI 0.73–1.59)] or retinol from food [RR = 0.74 (95% CI 0.50–1.08)]. All fracture risk were not associated with intake of vitamin A [RR = 0.91 (95% CI 0.82–1.02)] or retinol from food [RR = 0.91 (95% CI 0.82–1.01)].	[29]
Multicentre case cohort analysis	Men and women participating in Norwegian Epidemiologic Osteoporosis Studies between 1994–2001 (n = 21,774; aged 65–79 years)	Serum s-retinol concentration	Serum s-retinol concentration was not associated with hip fracture [HR = 0.99 (95% CI 0.88–1.10)].	[40]
Single-blind placebo-controlled trial	Healthy men (n = 80; aged 18–58 years)	Retinol palmitate (7576 μg)	Retinol palmitate did not affect bone-specific ALP, NTx and OCN.	[41]
Cross-sectional study	Postmenopausal women (n = 189, aged 50–75 years)	β-carotene intake	β-carotene intake was positively associated with T-score of lumbar spine, femoral neck, and total hip.	[23]
Cross-sectional study	Postmenopausal women (n = 160, aged 50–85 years)	Dietary pattern high in β-carotene and other nutrients	Dietary pattern high in β-carotene and other nutrients [OR = 0.08 (95% CI 0.02–0.15)] was positively associated with BMD at lumbar spine.	[25]
Case–control study	Elderly Chinese newly diagnosed with hip fractures and control participants in 2009–2013 (n = 1452)	β-carotene intake	Dietary intake of β-carotene [OR = 0.43 (95% CI 0.32–0.57)] was negatively associated with hip fracture risk.	[24]
Cross-sectional study	Subjects participating in KNHANES between 2008–2011 (n = 8022; aged 30–75 years)	β-carotene and β-cryptoxanthin intake	Intake of β-carotene was positively correlated with BMD at femoral neck, total hip, and whole body in postmenopausal women. Intake of β-cryptoxanthin was positively correlated with BMD at total hip in men and postmenopausal women. Subjects in highest daily intake of β-carotene [OR = 0.35 (95% CI 0.16–0.79)] and β-cryptoxanthin [OR = 0.76 (95% CI 0.59–0.97)] had lower risk of osteopenia.	[42]
Case–control study	Patients with hip fractures and age-matched controls (n = 2140, aged 55–80 years)	α-carotene, β-carotene and β-cryptoxanthin intake	Intake of α-carotene [OR = 0.45 (95% CI 0.30–0.66)], β-carotene [OR = 0.37 (95% CI 0.25–0.53)] and β-cryptoxanthin [OR = 0.40 (95% CI 0.28–0.56)] were negative associated with hip fracture risk.	[43]
Prospective cohort study	Chinese men and women recruited between 2008–2010 (n = 3169; aged 40–75 years)	Dietary consumption and serum level of β-carotene	Dietary intake of β-carotene was positively associated with BMD at total hip and femoral neck Serum levels of β-carotene and β-carotene-to-retinol ratio were positively associated with BMD at various sites.	[27]
Prospective cohort study	Non-pregnant women participating in Southampton Women’s Survey between 1998–2007 (n = 12,583; aged 20–34 years)	Maternal serum β-carotene concentration	Maternal serum β-carotene was positively associated with offspring total BMC and bone area at birth but not BMD or size-corrected BMC.	[34]
Cross-sectional study	Men and women participating in a cancer prevention programme between 1990–1996 (n = 998)	Plasma total carotene concentration	Plasma carotene concentration was negatively associated with risk of any fracture [HR plasma carotene = 0.88 (95% CI 0.68–1.14)].	[37]
Observational study	Postmenopausal women with and without osteoporosis (n = 90, aged ≥ 60 years)	Plasma α-carotene, β-carotene and retinol concentration	Plasma levels of α-carotene, β-carotene and retinol were lower in osteoporotics than in controls.	[44]
Cross-sectional study	Chinese men and women (n = 2831, aged 50–75 years)	Serum α-carotene concentration	Serum α-carotene concentration was positively associated with BMD at various skeletal sites (at whole body and hip regions).	[45]
Prospective cohort study	Postmenopausal women in the Nurses’ Health Study (n = 72,337; aged 34–77 years)	β-carotene intake	β-carotene intake from food source only [RR = 1.36 (95% CI 0.81–2.30)] as well as from food plus supplement [RR = 1.22 (95% CI 0.90–1.66)] was not associated with risk of hip fracture.	[32]
Population based longitudinal study	Men (n = 2322; aged 49–51 years)	β-carotene concentration	Serum β-carotene level was not associated with the risk of fracture [rate ratio = 0.95 (95% CI 0.81–1.11)].	[35]
Prospective, population-based cohort study	Dutch subjects participating in Rotterdam Study between 1990-1993 (n = 5288; aged ≥55 years)	Total β-carotene intake	No interactions between dietary β-carotene intake and BMD.	[11]

List of abbreviations: 1,25(OH)_2_D_3_: 1,25-dihydoxyvitamin D_3_; ALP: alkaline phosphatase; BMD: bone mineral density; IU: international units; HR: hazard ratio; KNHANES: Korea National Health & Nutrition Examination Survey; NHANES: National Health & Nutrition Examination Survey; NTx: N-telopeptide of type-1 collagen; OCN: osteocalcin; OR: odds ratio; RBP4: retinol binding protein 4; RR: relative risk; TTR: transthyretin.

**Table 3:** Summary on the effects of vitamin A and provitamin A on bone cells.

Type of Cell	Treatment & Concentration	Findings	Reference
MC3T3-E1 cells	Retinol (1–100 nM), retinoic acid (1–100 nM), or β-carotene (0.1–10 µM)	All treatments increased osteoblast differentiation, ALP activity and OPN expression	[53]
Primary human osteoblasts	Retinoic acid (4-400 nM)	Retinoic acid reduced calcium deposition	[54]
MC3T3-E1 cells	Retinoic acid (400 nM)	Retinoic acid reduced cell proliferation, ALP, OCN, Runx2, OSX, Phex, SOST, and FGF-23, but increased RANKL and Dmp1. Retinoic acid suppressed osteoblast mineralisation via RAR signalling.	
MC3T3-E1 cells	Combination of β-carotene and isoflavones (0.1–10 µM)	β-carotene and isoflavones increased ALP activity β-carotene enhanced the expression of Runx-2, ALP, and OPN. β-carotene enhanced early osteoblastic differentiation via RAR signalling.	[55]
Bone marrow-derived monocytes/macrophages stimulated by RANKL	β-carotene (0.4–0.6 µM)	β-carotene inhibited cell viability, promoted LDH release, reduced density of TRAP-positive areas, osteoclast numbers and resorption pit formation. β-carotene attenuated NF-κB activation but had no effect on MAPK pathway. β-carotene downregulated NFATc1, c-Fos, and CTSK	[10]
Primary osteoblastic cells isolated from newborn mouse calvariae stimulated by LPS	β-cryptoxanthin (5–10 µM)	β-cryptoxanthin reduced COX-2, mPGES1, PGE_2_ and RANKL.	[9]
RAW264.7 cells stimulated by RANKL	β-cryptoxanthin (5–10 µM)	β-cryptoxanthin suppressed NF-κB activation and reduced CTSK expression.	

List of abbreviations: ALP: alkaline phosphatase; c-FOS: Fos proto-oncogene; COX-2: cyclooxygenase-2; CTSK: Cathepsin K; Dmp1: dentin matrix protein 1; FGF-23: fibroblast growth factor 23; LDH: lactate dehydrogenase; LPS: lipopolysaccharide; MAPK: mitogen-activated protein kinase; mPGES1: membrane-bound PGE synthase-1; NFATc1: nuclear factor of activated T-cell cytoplasmic 1; NF-κB: nuclear factor kappa B; OCN: osteocalcin; OPN: osteopontin; OSX: osterix; PGE_2_: prostaglandin E2; Phex: phosphate regulating endopeptidase homolog X-linked; RANKL: receptor activator of nuclear factor-kappa B ligand; RAR: retinoic acid receptor; Runx2: runt-related transcription factor 2; SOST: sclerostin; TRAP: tartrate-resistant acid phosphatase.
